# Supplementary material for: Genetic Variants in the ABCB1 and ABCG2 Gene Drug Transporters Involved in Gefitinib-Associated Adverse Reaction: A Systematic Review and Meta-Analysis
Source: Genes (Basel). 2024 May 7;15(5):591. doi: 10.3390/genes15050591 (PMC11120674; doi:10.3390/genes15050591)
Supplement: Supplementary file 1 [file genes-15-00591-s001.zip › genes-2981512-supplementary/genes-2981512-supplementary Tables S2-S4 and Figures S1-S6.pdf]

(**a**)

Study or Subgroup	CC Events	CC Total	CT + TT Events	CT + TT Total	Weight	Odds Ratio M-H, Fixed, 95% CI
Ma 2016 [23]	5	8	12	26	25.6%	1.94 [0.38, 9.88]
Tamura 2012 [31]	7	23	16	60	74.4%	1.20 [0.42, 3.46]
**Total (95% CI)**		**31**		**86**	**100.0%**	**1.39 [0.58, 3.35]**
Total events	12		28			

Heterogeneity: Chi² = 0.24, df = 1 (P = 0.63); I² = 0%
Test for overall effect: Z = 0.74 (P = 0.46)

Odds Ratio
M-H, Fixed, 95% CI

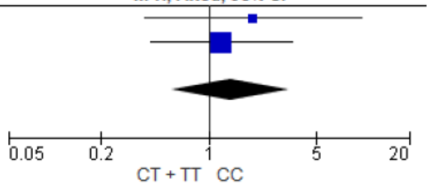

(**b**)

Study or Subgroup	CC Events	CC Total	CT + TT Events	CT + TT Total	Weight	Odds Ratio M-H, Fixed, 95% CI
Ma 2016 [23]	1	7	3	15	50.7%	0.67 [0.06, 7.85]
Tamura 2012 [31]	1	23	3	60	49.3%	0.86 [0.09, 8.75]
**Total (95% CI)**		**30**		**75**	**100.0%**	**0.76 [0.14, 4.14]**
Total events	2		6			

Heterogeneity: Chi² = 0.02, df = 1 (P = 0.88); I² = 0%
Test for overall effect: Z = 0.31 (P = 0.75)

Odds Ratio
M-H, Fixed, 95% CI

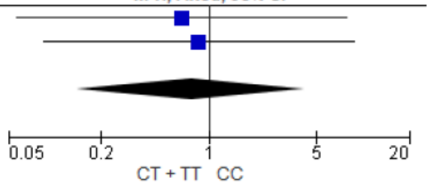

(**c**)

Study or Subgroup	CC Events	CC Total	CT + TT Events	CT + TT Total	Weight	Odds Ratio M-H, Fixed, 95% CI
Ma 2016 [23]	6	9	5	8	23.4%	1.20 [0.16, 8.80]
Tamura 2012 [31]	3	23	12	60	76.6%	0.60 [0.15, 2.36]
**Total (95% CI)**		**32**		**68**	**100.0%**	**0.74 [0.24, 2.24]**
Total events	9		17			

Heterogeneity: Chi² = 0.32, df = 1 (P = 0.57); I² = 0%
Test for overall effect: Z = 0.53 (P = 0.59)

Odds Ratio
M-H, Fixed, 95% CI

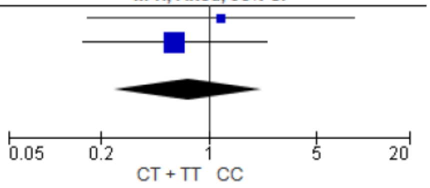

Figure S1. Forest plots of association between adenosine triphosphate-binding cassette subfamily B member 1 (*ABCB1*) gene (rs1045642) genetic variant and adverse drug reaction severity (grade 0 versus grade 0 + 1). (**a**) skin rash (CC versus. CT + TT); (**b**) diarrhea (CC versus CT + TT); and (**c**) liver dysfunction (CC versus CT + TT) [23,31]. CI, confidence interval; M-H, Mentel-Haenszel; I², heterogeneity; Chi², Chi-square test for heterogeneity.

(**a**)

Study or Subgroup	CC Events	CC Total	CA + AA Events	CA + AA Total	Weight	Odds Ratio M-H, Fixed, 95% CI
Lemos 2020 [21]	21	78	1	9	10.2%	2.95 [0.35, 25.01]
Ma 2016 [23]	8	21	9	27	37.8%	1.23 [0.37, 4.05]
Tamura 2012 [31]	14	45	9	38	52.1%	1.46 [0.55, 3.87]
**Total (95% CI)**		**144**		**74**	**100.0%**	**1.52 [0.75, 3.07]**
Total events	43		19			

Heterogeneity: $Chi^2 = 0.50$, df = 2 (P = 0.78); $I^2 = 0\%$
Test for overall effect: Z = 1.17 (P = 0.24)

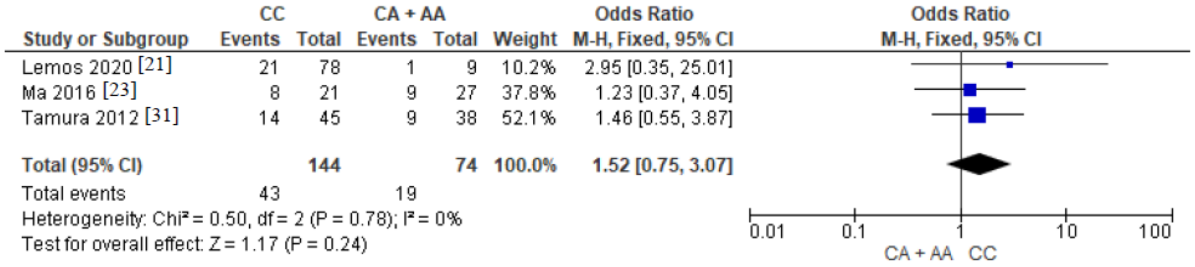

(**b**)

Study or Subgroup	CC Events	CC Total	CA + AA Events	CA + AA Total	Weight	Odds Ratio M-H, Fixed, 95% CI
Lemos 2020 [21]	6	76	0	9	29.3%	1.75 [0.09, 33.64]
Ma 2016 [23]	2	11	2	27	34.2%	2.78 [0.34, 22.75]
Tamura 2012 [31]	3	45	1	38	36.5%	2.64 [0.26, 26.52]
**Total (95% CI)**		**132**		**74**	**100.0%**	**2.43 [0.60, 9.89]**
Total events	11		3			

Heterogeneity: $Chi^2 = 0.07$, df = 2 (P = 0.97); $I^2 = 0\%$
Test for overall effect: Z = 1.24 (P = 0.22)

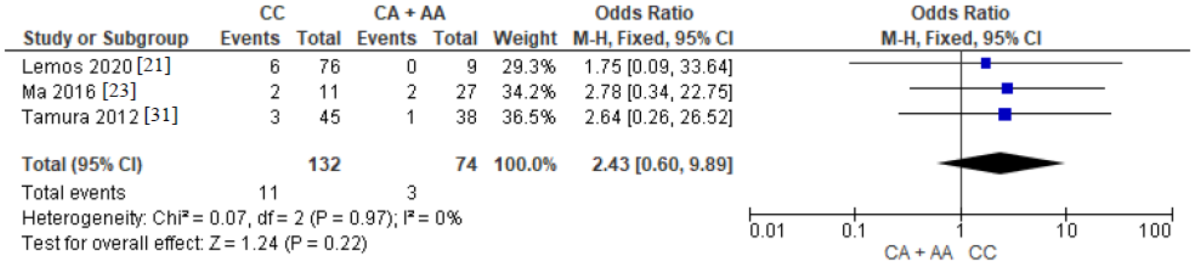

(**c**)

Study or Subgroup	CC Events	CC Total	CA + AA Events	CA + AA Total	Weight	Odds Ratio M-H, Fixed, 95% CI
Ma 2016 [23]	1	22	4	30	34.1%	0.31 [0.03, 2.98]
Tamura 2012 [31]	8	45	7	38	65.9%	0.96 [0.31, 2.94]
**Total (95% CI)**		**67**		**68**	**100.0%**	**0.74 [0.28, 1.95]**
Total events	9		11			

Heterogeneity: $Chi^2 = 0.77$, df = 1 (P = 0.38); $I^2 = 0\%$
Test for overall effect: Z = 0.61 (P = 0.54)

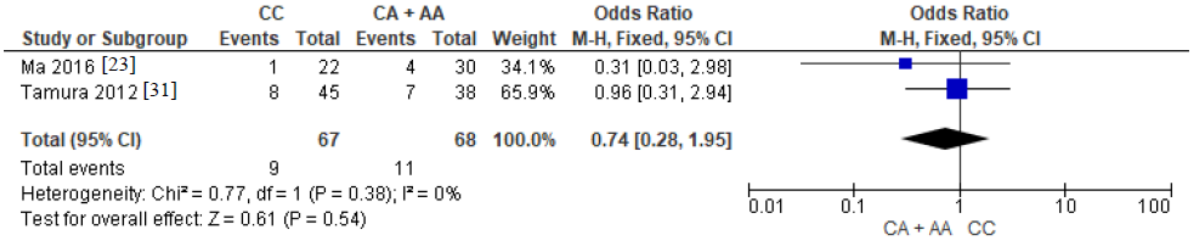

Figure S2. Forest association plots between adenosine triphosphate-binding cassette subfamily G member 2 (*ABCG2*) gene (rs2231142) genetic variant and adverse drug reaction severity (grade ≥ 2 versus 0 + 1). (**a**) skin rash (CC versus CA + AA); (**b**) diarrhea (CC versus CA + AA); and (**c**) liver dysfunction (CC versus CA + AA) [21,21,31]. CI, confidence interval; M-H, Mentel-Haenszel; I^2, heterogeneity; Chi^2, Chi-square test for heterogeneity.

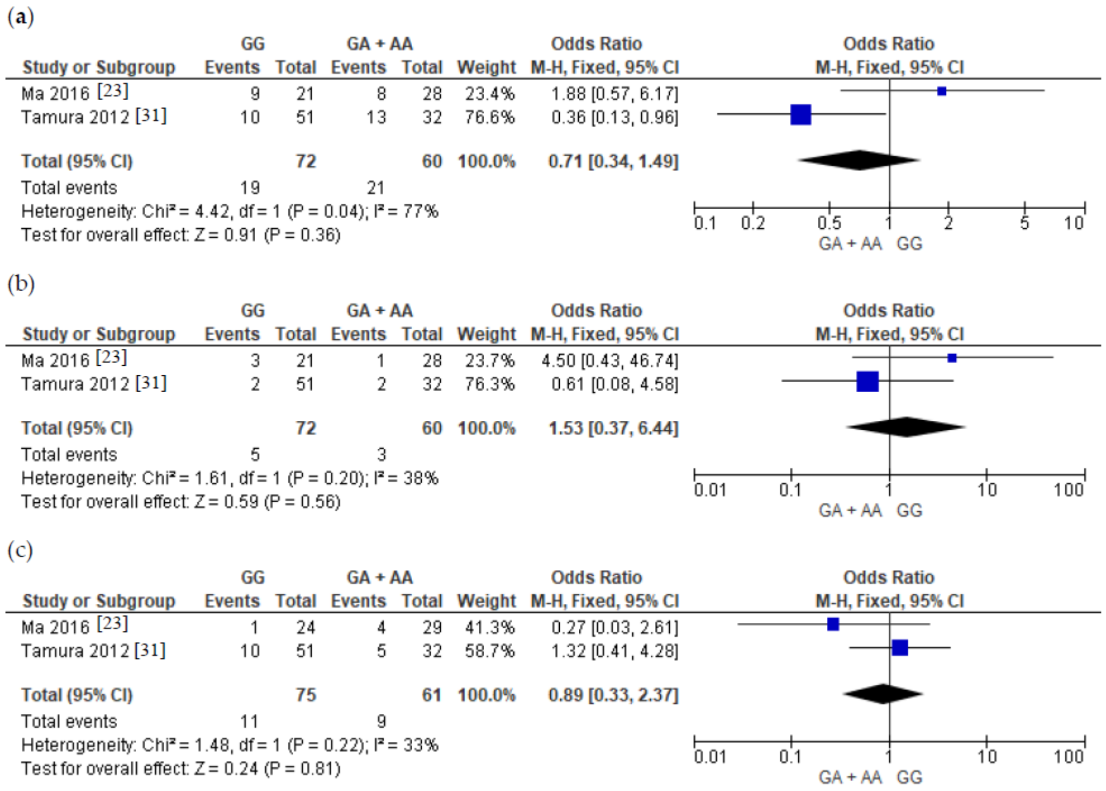

Figure S3. Forest association plots between adenosine triphosphate-binding cassette subfamily G member 2 (*ABCG2*) gene (rs2231137) genetic variant and adverse drug reaction severity (grade 1 versus grade ≥ 2). (**a**) skin rash (GG versus GA + AA); (**b**) diarrhea (GG versus GA + AA); and (**c**) liver dysfunction (GG versus GA + AA) [23,31]. CI, confidence interval; M-H, Mentel-Haenszel; I^2, heterogeneity; Chi^2, Chi-square test for heterogeneity.

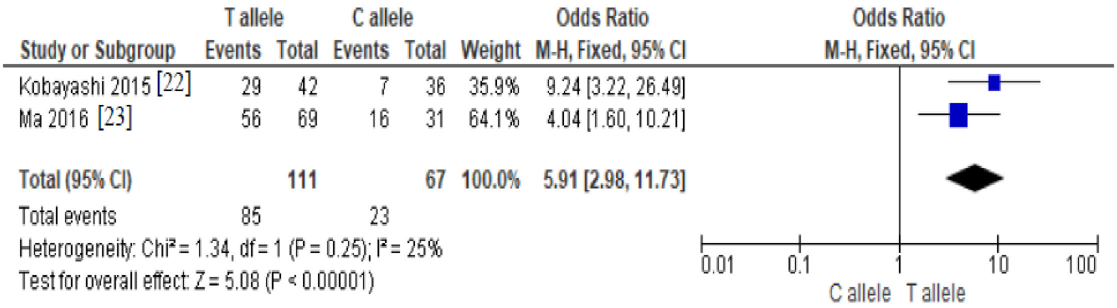

Figure S4. Forest plots of association between adenosine triphosphate-binding cassette subfamily B member 1 (*ABCB1*) gene (rs1128503) genetic variant and adverse drug reaction (grade 0 versus grade ≥ 1). (**a**) skin rash (T allele versus C allele) [22-23]. CI, confidence interval; M-H, Mentel-Haenszel; I^2, heterogeneity; Chi^2, Chi-square test for heterogeneity.

	T allele		C allele			Odds Ratio
Study or Subgroup	Events	Total	Events	Total	Weight	M-H, Fixed, 95% CI
Kobayashi 2015 [22]	18	28	12	34	26.9%	3.30 [1.16, 9.38]
Ma 2016 [23]	20	41	24	55	73.1%	1.23 [0.55, 2.77]
**Total (95% CI)**		**69**		**89**	**100.0%**	**1.79 [0.95, 3.36]**
Total events	38		36			

Heterogeneity: $Chi^2 = 2.14$, df = 1 (P = 0.14); $I^2 = 53\%$

Test for overall effect: Z = 1.80 (P = 0.07)

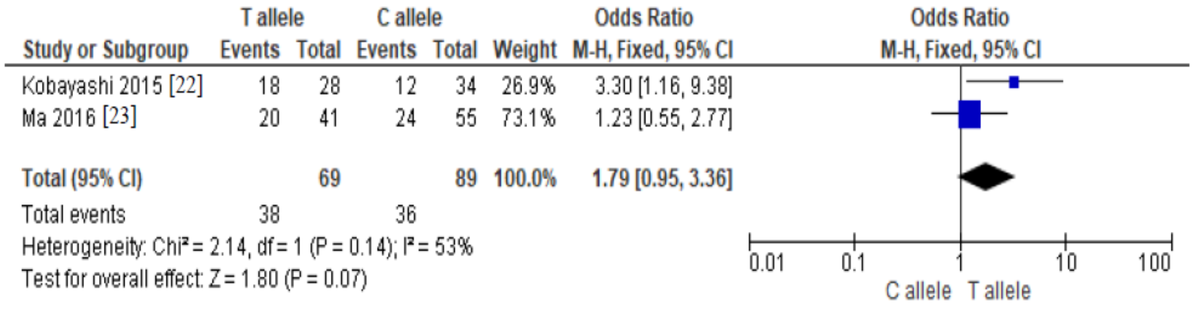

Figure S5. Forest plots of association between adenosine triphosphate-binding cassette subfamily B member 1 (*ABCB1*) gene (rs1045642) genetic variant and adverse drug reaction (grade 0 versus grade ≥ 1) (**a**) skin rash (T allele versus C allele) [22-23]. CI, confidence interval; M-H, Mentel-Haenszel; I^2, heterogeneity; Chi^2, Chi-square test for heterogeneity.

(a)

Study or Subgroup	GG Events	GG Total	GT+TT+TA+AA Events	GT+TT+TA+AA Total	Weight	Odds Ratio M-H, Fixed, 95% CI
Kobayashi 2015 [22]	0	3	18	25	63.8%	0.06 [0.00, 1.26]
Ma 2016 [23]	4	6	22	30	36.2%	0.73 [0.11, 4.77]
**Total (95% CI)**		**9**		**55**	**100.0%**	**0.30 [0.07, 1.21]**
Total events	4		40			

Heterogeneity: Chi² = 1.95, df = 1 (P = 0.16); I² = 49%
Test for overall effect: Z = 1.69 (P = 0.09)

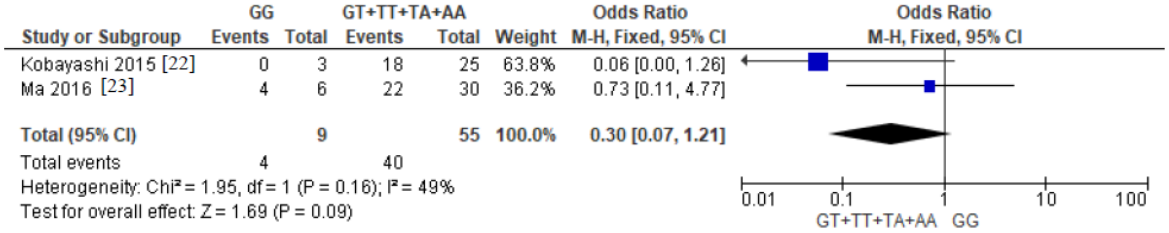

(b)

Study or Subgroup	GG Events	GG Total	GT+TT+TA+AA Events	GT+TT+TA+AA Total	Weight	Odds Ratio M-H, Fixed, 95% CI
Kobayashi 2015 [22]	1	3	14	25	60.7%	0.39 [0.03, 4.92]
Ma 2016 [23]	4	6	12	31	39.3%	3.17 [0.50, 20.04]
**Total (95% CI)**		**9**		**56**	**100.0%**	**1.48 [0.37, 5.92]**
Total events	5		26			

Heterogeneity: Chi² = 1.71, df = 1 (P = 0.19); I² = 42%
Test for overall effect: Z = 0.56 (P = 0.58)

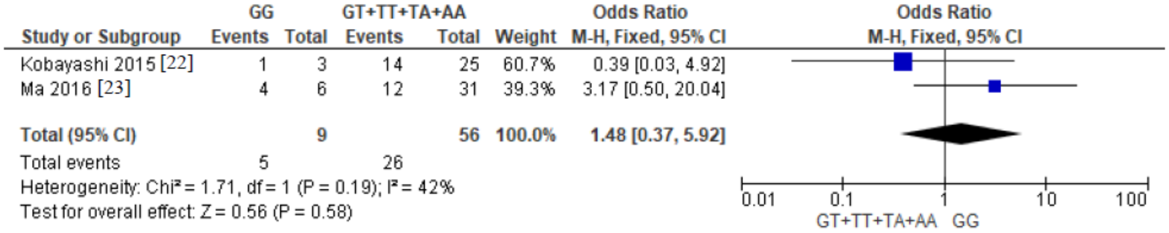

(c)

Study or Subgroup	GG Events	GG Total	GT+TT+TA+AA Events	GT+TT+TA+AA Total	Weight	Odds Ratio M-H, Fixed, 95% CI
Kobayashi 2015 [22]	1	3	7	25	26.1%	1.29 [0.10, 16.54]
Ma 2016 [23]	0	5	9	30	73.9%	0.21 [0.01, 4.11]
**Total (95% CI)**		**8**		**55**	**100.0%**	**0.49 [0.08, 3.04]**
Total events	1		16			

Heterogeneity: Chi² = 0.87, df = 1 (P = 0.35); I² = 0%
Test for overall effect: Z = 0.77 (P = 0.44)

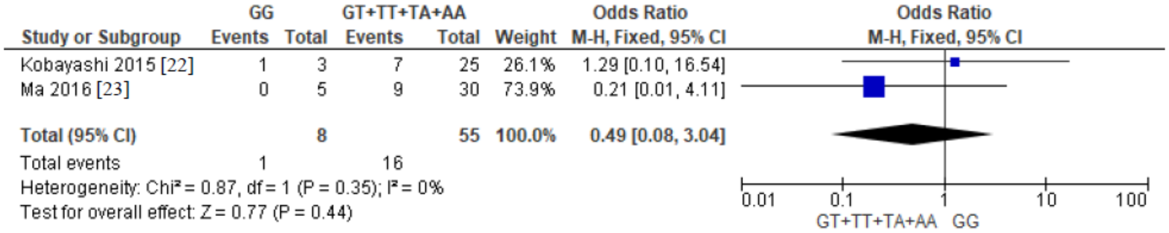

Figure S6. Forest plots of association between adenosine triphosphate-binding cassette subfamily B member 1 (*ABCB1*) gene (rs2032582) genetic variant and adverse drug reaction (grade 0 versus grade ≥ 1). (**a**) skin rash (GG versus. GT + TT + TA+ AA); (**b**) diarrhea (GG versus. GT + TT + TA+ AA); and (**c**) liver dysfunction (GG versus. GT + TT + TA+ AA) [22-23]. CI, confidence interval; M-H, Mentel-Haenszel; I^2, heterogeneity; Chi^2, Chi-square test for heterogeneity.

Table S2. Excluded Studies

Absolut Number	Reason for exclusion	Authors, year	Title	Reference
1	Wrong population	Wan Z, et *al.*, 2020	Determinants of gefitinib pharmacokinetics in healthy Chinese male subjects: A pharmacogenomics study of cytochrome p450 enzymes and transporters	[49]
2	Wrong, comparator	Tiseo M, *et al.*, 2010.	Predictors of gefitinib outcomes in advanced non-small cell lung cancer (NSCLC): Study of a comprehensive panel of molecular markers.	[50]
3	Wrong comparator	Kobayashi H, *et al.*, 2016.	Effects of polymorphisms in CYP2D6 and ABC transporters and side effects induced by gefitinib on the pharmacokinetics of the gefitinib metabolite, O-desmethyl gefitinib.	[51]
4	Wrong comparator.	Cho BC *et al.*, 2019.	Osimertinib versus standard of care EGFR TKI as first-line treatment in patients with EGFRm advanced NSCLC: FLAURA Asian subset.	[52]
5	Wrong comparator.	Mou K, *et al.*, 2016.	Relationship between miR-7 expression and treatment outcomes with gefitinib in non-small cell lung cancer.	[53]
6	Wrong outcome	Ruan Y, *et al.*, 2016.	Genetic association of curative and adverse reactions to tyrosine kinase inhibitors in Chinese advanced non-small cell lung cancer patients	[54]
7	Wrong publication type	Akasaka K, *et al.* 2010.	Impact of functional ABCG2 polymorphisms on the adverse effects of gefitinib in Japanese patients with non-small-cell lung cancer	[55]
8	Wrong publication type	Cusatis G, *et al.* 2006.	Pharmacogenetics of ABCG2 and adverse reactions to gefitinib.	[46]
9	Only abstract.	He Y, *et al.*, 2017.	Comparing EGFR-TKI with EGFR-TKI plus chemotherapy as 1st line treatment in advanced NSCLC patients with both mutated EGFR and Bim polymorphism	[56]
10	Only abstract.	Jeon EK, *et al.*, 2010.	The association between SNP of EGFR and response to EGFR-TKIs according to EGFR mutation in non-small cell lung cancer	[57]
11	Only abstract and metabolizer gene	Suzumura T, *et al.*, 2012.	Reduced CYP2D6 function potentiates the gefitinib-induced rash in patients with non-small cell lung cancer	[58]
12	Only abstract.	Xin S, *et al.*, 2015.	Correlation of polymorphisms of JAK and STAT with gefitinib-induced hepatotoxicity in patients with non-small cell lung cancer.	[59]
13	*ABCB1* or *ABCB2* gene not involved in outcome	Takimoto T, *et al.*, 2013	Polymorphisms of *CYP2D6* Gene and Gefitinib-Induced Hepatotoxicity	[14]

14	*ABCB1* or *ABCB2* gene not involved in outcome	Giovanetti E, *et al.*, 2010.	Association of polymorphisms in AKT1 and EGFR with clinical outcome and toxicity in non-small cell lung cancer patients treated with gefitinib	[60]
15	*ABCB1* or *ABCB2* gene not involved in outcome	Gregorc V, *et al.*, 2008.	Germline polymorphisms in EGFR and survival in patients with lung cancer receiving gefitinib	[61]
16	*ABCB1* or *ABCB2* gene not involved in outcome	Huang CL, *et al.*, 2009.	EGFR intron 1 dinucleotide repeat polymorphism is associated with the occurrence of skin rash with gefitinib treatment	[62]
17	*ABCB1* or *ABCB2* gene not involved in outcome	Sugiyama E, *et al.*, 2015	Impact of single nucleotide polymorphisms on severe hepatotoxicity induced by EGFR tyrosine kinase inhibitors in patients with non-small cell lung cancer harboring EGFR mutations	[15]
18	*ABCB1* or *ABCB2* gene not involved in outcome	Giovanetti E, *et al.*, 2011.	Influence of polymorphism on EGFR target therapy in non-small-cell lung cancer	[63]
19	*ABCB1* or *ABCB2* gene not involved in outcome	Xin S, *et al.*, 2019.	Polymorphisms of NF-kB pathway gene influence adverse drug reactions of gefitinib in NSCLC patients	[64]
20	*ABCB1* or *ABCB2* gene not involved in outcome	Liu G, *et al.*, 2008.	Epidermal growth factor receptor polymorphisms and clinical outcomes in non-small-cell lung cancer patients treated with gefitinib	[65]

Table S3. Allelic description for meta-analysis (grades ≥ 2 versus grades 0 + 1).

***ABCB1* gene - db./ID 1045642 (3435C>T)**						
**Tamura, et al., 2012 [31]**						
ADRs	**Skin Rash**		**Diarrhea**		**Liver Dysfunction**	
Alleles	CC	CC + CT	CC	CT + TT	CC	CT + TT
Grade 0 -1	16	44	22	57	20	48
Grade ≥ 2	7	16	1	3	3	12
Total	23	60	23	60	23	60
**Ma, et al., 2017 [23]**						
ADRs	**Skin Rash**		**Diarrhea**		**Liver Dysfunction**	
Alleles	CC	CT + TT	CC	CT + TT	CC	CT + TT
Grade 0 -1	9	22	13	31	15	31
Grade ≥ 2	5	12	1	3	1	4
Total	14	34	14	34	16	35
***ABCG2* gene – db./ID 2231142 (421C>A)**						
**Tamura, et al., 2012 [31]**						
ADRs	**Skin Rash**		**Diarrhea**		**Liver Dysfunction**	
Alleles	CC	CA + AA	CC	CA + AA	CC	CA + AA
Grade 0 -1	31	29	42	37	37	31
Grade ≥ 2	14	9	3	1	8	7
Total	45	38	45	38	45	38
**Ma, et al., 2017 [23]**						
ADRs	**Skin Rash**		**Diarrhea**		**Liver Dysfunction**	
Alleles	CC	CA + AA	CC	CA + AA	CC	CA + AA
Grade 0 -1	13	18	9	25	21	26
Grade ≥ 2	8	9	2	2	1	4
Total	21	27	11	27	22	30
**Study Lemos et al., 2020 [21]**						
ADRs	**Skin Rash**		**Diarrhea**		**Liver Dysfunction**	
Alleles	CC	CA + AA	CC	CA + AA	CC	CA + AA
Grade 0 -1	57	8	70	9	-	-
Grade ≥ 2	21	1	6	0	-	-
Total	78	9	76	9	-	-
***ABCG2* gene – db./ID 2231137**						
**Tamura, et al., 2012 [31]**						
ADRs	**Skin Rash**		**Diarrhea**		**Liver Dysfunction**	
Alleles	GG	GA + AA	GG	GA + AA	GG	GA + AA
Grade 0 -1	41	19	49	30	41	27
Grade ≥ 2	10	13	2	2	10	5
Total	51	32	51	32	51	32
**Ma, et al., 2017 [23]**						
ADRs	**Skin Rash**		**Diarrhea**		**Liver Dysfunction**	
Alleles	GG	GA+AA	GG	GA+AA	GG	GA+AA
Grade 0 -1	12	20	18	27	23	25
Grade ≥ 2	9	8	3	1	1	4
Total	21	28	21	28	24	29

ADR: adverse drug reaction; *ABCB1*: adenosine triphosphate-binding cassette subfamily B member 1; and *ABCG2*: adenosine triphosphate-binding cassette subfamily G member 2

Table S4. Allelic description for meta-analysis (grades 0 versus grades ≥ 1).

***ABCB1* gene - db./ID 1128503 (1236C>T)**									
**Kobayashi *et al.*, 2015 [22]**									
ADRs	Skin Rash			Diarrhea			Liver Dysfunction		
Alleles	CC	CT	TT	CC	CT	TT	CC	CT	TT
Grade 0	3	3	4	3	2	8	3	3	7
Grade ≥ 1	1	5	12	1	6	8	1	5	9
Total	4	8	16	4	8	16	4	8	16
**Ma, et al., 2017 [23]**									
ADRs	Skin Rash			Diarrhea			Liver Dysfunction		
Alleles	CC	CT	TT	CC	CT	TT	CC	CT	TT
Grade 0	4	7	3	4	14	9	4	20	20
Grade ≥ 1	2	12	22	2	5	16	3	4	4
Total	6	19	25	6	19	25	6	24	24
***ABCB1* gene - db./ID 1045642 (3435C>T)**									
**Kobayashi *et al.*, 2015 [22]**									
ADRs	Skin Rash			Diarrhea			Liver Dysfunction		
Alleles	CC	CT	TT	CC	CT	TT	CC	CT	TT
Grade 0	5	4	2	7	8	1	5	7	2
Grade ≥ 1	5	10	5	3	6	6	5	7	5
Total	10	14	7	10	14	7	10	14	7
**Ma, et al., 2017 [23]**									
ADRs	Skin Rash			Diarrhea			Liver Dysfunction		
Alleles	CC	CT	TT	CC	CT	TT	CC	CT	TT
Grade 0	6	8	0	7	17	2	12	22	6
Grade ≥ 1	8	19	7	7	10	5	4	7	0
Total	14	27	7	14	27	7	14	29	6
***ABCB1* gene - db./ID 2032582 (2677G>T/A)**									
**Kobayashi *et al.*, 2015 [22]**									
ADRs	Skin Rash			Diarrhea			Liver Dysfunction		
Alleles	GG	GT	TT+TA+AA	GG	GT	TT+TA+AA	GG	GT	TT+TA+AA
Grade 0	3	5	2	2	8	3	2	6	5
Grade ≥ 1	0	9	9	1	6	8	1	8	6
Total	3	14	11	3	14	11	3	14	11
**Ma, et al., 2017 [23]**									
ADRs	Skin Rash			Diarrhea			Liver Dysfunction		
Alleles	GG	GT	TT+TA+AA	GG	GT	TT+TA+AA	GG	GT	TT+TA+AA
Grade 0	2	7	1	2	11	8	5	16	7
Grade ≥ 1	4	14	8	4	11	1	0	7	2
Total	6	21	9	6	22	9	5	23	9
***ABCG2* gene – db./ID 2231142 (421C>A)**									
**Kobayashi *et al.*, 2015 [22]**									
ADRs	Skin Rash			Diarrhea			Liver Dysfunction		
Alleles	CC	CA + AA		CC	CA + AA		CC	CA + AA	
Grade 0	4	7		7	9		8	6	
Grade ≥ 1	12	8		9	6		8	9	
Total	16	15		16	15		16	15	

**Ma, et al., 2017 [23]**						
ADRs	Skin Rash		Diarrhea		Liver Dysfunction	
Alleles	CC	CA + AA	CC	CA + AA	CC	CA + AA
Grade 0	4	9	0	16	20	22
Grade ≥ 1	17	18	11	11	2	8
Total	21	27	11	27	22	30
**Lemos et al., 2020 [21]**						
ADRs	Skin Rash		Diarrhea		Liver Dysfunction	
Alleles	CC	CA + AA	CC	CA + AA	CC	CA + AA
Grade 0	36	5	45	7	-	-
Grade ≥ 1	42	4	31	2	-	-
Total	78	9	76	9	-	-

ADR: adverse drug reaction; *ABCB1*: adenosine triphosphate-binding cassette subfamily B member 1; and *ABCG2*: adenosine triphosphate-binding cassette subfamily G member 2
