# Supplementary material for: Genetic Variants in the ABCB1 and ABCG2 Gene Drug Transporters Involved in Gefitinib-Associated Adverse Reaction: A Systematic Review and Meta-Analysis
Source: Genes (Basel). 2024 May 7;15(5):591. doi: 10.3390/genes15050591 (PMC11120674; doi:10.3390/genes15050591)
Supplement: Supplementary file 1 [file genes-15-00591-s001.zip › genes-2981512-supplementary/genes-2981512-supplementary-Table S1.pdf]

Table S1. Search Strategy

**Databases**	**Search Strategy**	**January, 10, 2024**
PUBMED	((((((MicroRNAs[MeSH Terms]) OR (MicroRNAs[Title/Abstract] OR "MicroRNA"[Title/Abstract] OR miRNAs[Title/Abstract] OR "Micro RNA"[Title/Abstract] OR "RNA, Micro"[Title/Abstract] OR miRNA[Title/Abstract] OR "Primary MicroRNA"[Title/Abstract] OR "MicroRNA, Primary"[Title/Abstract] OR "Primary miRNA"[Title/Abstract] OR "miRNA, Primary"[Title/Abstract] OR "pri-miRNA"[Title/Abstract] OR "pri miRNA"[Title/Abstract] OR "RNA, Small Temporal"[Title/Abstract] OR "Temporal RNA, Small"[Title/Abstract] OR stRNA[Title/Abstract] OR "Small Temporal RNA"[Title/Abstract] OR "pre-miRNA"[Title/Abstract] OR "pre miRNA"[Title/Abstract])) OR ((Epigenomics[MeSH Terms]) OR (Epigenomics[Title/Abstract] OR Epigenomic[Title/Abstract] OR Epigenetics[Title/Abstract] OR Epigenetic[Title/Abstract]))) OR ((Polymorphism, Single Nucleotide[MeSH Terms]) OR ("Polymorphism, Single Nucleotide"[Title/Abstract] OR "Nucleotide Polymorphism, Single"[Title/Abstract] OR "Nucleotide Polymorphisms, Single"[Title/Abstract] OR "Polymorphisms, Single Nucleotide"[Title/Abstract] OR "Single Nucleotide Polymorphisms"[Title/Abstract] OR "SNPs"[Title/Abstract] OR "Single Nucleotide Polymorphism"[Title/Abstract]))) OR ((Polymorphism, Genetic[MeSH Terms]) OR ("Polymorphism, Genetic"[Title/Abstract] OR "Polymorphisms, Genetic"[Title/Abstract] OR "Genetic Polymorphisms"[Title/Abstract] OR "Genetic Polymorphism"[Title/Abstract] OR "Polymorphism (Genetics)"[Title/Abstract] OR "Polymorphisms (Genetics)"[Title/Abstract]))) AND ((((Carcinoma, Non-Small-Cell Lung[MeSH Terms]) OR ("Carcinoma, Non-Small-Cell Lung"[Title/Abstract] OR "Carcinoma, Non Small Cell Lung"[Title/Abstract] OR "Carcinomas, Non-Small-Cell Lung"[Title/Abstract] OR "Lung Carcinoma, Non-Small-Cell"[Title/Abstract] OR "Lung Carcinomas, Non-Small-Cell"[Title/Abstract] OR "Non-Small-Cell Lung Carcinomas"[Title/Abstract] OR "Nonsmall Cell Lung Cancer"[Title/Abstract] OR "Non-Small-Cell Lung Carcinoma"[Title/Abstract] OR "Non Small Cell Lung Carcinoma"[Title/Abstract] OR "Carcinoma, Non-Small Cell Lung"[Title/Abstract] OR "Non-Small Cell Lung Cancer"[Title/Abstract])) OR ((Lung Neoplasms[MeSH Terms]) OR ("Lung Neoplasms"[Title/Abstract] OR "Pulmonary Neoplasms"[Title/Abstract] OR "Neoplasms,	280

	Lung"[Title/Abstract] OR "Lung Neoplasm"[Title/Abstract] OR "Neoplasm, Lung"[Title/Abstract] OR "Neoplasms, Pulmonary"[Title/Abstract] OR "Neoplasm, Pulmonary"[Title/Abstract] OR "Pulmonary Neoplasm"[Title/Abstract] OR "Lung Cancer"[Title/Abstract] OR "Cancer, Lung"[Title/Abstract] OR "Cancers, Lung"[Title/Abstract] OR "Lung Cancers"[Title/Abstract] OR "Pulmonary Cancer"[Title/Abstract] OR "Cancer, Pulmonary"[Title/Abstract] OR "Cancers, Pulmonary"[Title/Abstract] OR "Pulmonary Cancers"[Title/Abstract] OR "Cancer of the Lung"[Title/Abstract] OR "Cancer of Lung"[Title/Abstract]))) OR ("Lung Tumor"))) AND ((Gefitinib[MeSH Terms]) OR (Gefitinib[Title/Abstract] OR "N-(3-Chloro-4-fluorophenyl)-7-methoxy-6-(3-(4-morpholinyl)propoxy)-4-quinazolinamide"[Title/Abstract] OR Iressa[Title/Abstract] OR ZD1839[Title/Abstract] OR "ZD 1839"[Title/Abstract])) Filters: English, Portuguese, Spanish Sort by: Publication Date	
PUBMED PMC	((((((MicroRNAs[MeSH Terms]) OR (MicroRNAs[Title/Abstract] OR "MicroRNA"[Title/Abstract] OR miRNAs[Title/Abstract] OR "Micro RNA"[Title/Abstract] OR "RNA, Micro"[Title/Abstract] OR miRNA[Title/Abstract] OR "Primary MicroRNA"[Title/Abstract] OR "MicroRNA, Primary"[Title/Abstract] OR "Primary miRNA"[Title/Abstract] OR "miRNA, Primary"[Title/Abstract] OR "pri-miRNA"[Title/Abstract] OR "pri miRNA"[Title/Abstract] OR "RNA, Small Temporal"[Title/Abstract] OR "Temporal RNA, Small"[Title/Abstract] OR stRNA[Title/Abstract] OR "Small Temporal RNA"[Title/Abstract] OR "pre-miRNA"[Title/Abstract] OR "pre miRNA"[Title/Abstract])) OR ((Epigenomics[MeSH Terms]) OR (Epigenomics[Title/Abstract] OR Epigenomic[Title/Abstract] OR Epigenetics[Title/Abstract] OR Epigenetic[Title/Abstract]))) OR ((Polymorphism, Single Nucleotide[MeSH Terms]) OR ("Polymorphism, Single Nucleotide"[Title/Abstract] OR "Nucleotide Polymorphism, Single"[Title/Abstract] OR "Nucleotide Polymorphisms, Single"[Title/Abstract] OR "Polymorphisms, Single Nucleotide"[Title/Abstract] OR "Single Nucleotide Polymorphisms"[Title/Abstract] OR "SNPs"[Title/Abstract] OR "Single Nucleotide Polymorphism"[Title/Abstract]))) OR ((Polymorphism, Genetic[MeSH Terms]) OR ("Polymorphism, Genetic"[Title/Abstract] OR "Polymorphisms, Genetic"[Title/Abstract] OR "Genetic Polymorphisms"[Title/Abstract] OR "Genetic Polymorphism"[Title/Abstract] OR "Polymorphism (Genetics)"[Title/Abstract] OR "Polymorphisms (Genetics)"[Title/Abstract]))) AND ((((Carcinoma, Non-	78

	Small-Cell Lung[MeSH Terms]) OR ("Carcinoma, Non-Small-Cell Lung"[Title/Abstract] OR "Carcinoma, Non Small Cell Lung"[Title/Abstract] OR "Carcinomas, Non-Small-Cell Lung"[Title/Abstract] OR "Lung Carcinoma, Non-Small-Cell"[Title/Abstract] OR "Lung Carcinomas, Non-Small-Cell"[Title/Abstract] OR "Non-Small-Cell Lung Carcinomas"[Title/Abstract] OR "Nonsmall Cell Lung Cancer"[Title/Abstract] OR "Non-Small-Cell Lung Carcinoma"[Title/Abstract] OR "Non Small Cell Lung Carcinoma"[Title/Abstract] OR "Carcinoma, Non-Small Cell Lung"[Title/Abstract] OR "Non-Small Cell Lung Cancer"[Title/Abstract])) OR ((Lung Neoplasms[MeSH Terms]) OR ("Lung Neoplasms"[Title/Abstract] OR "Pulmonary Neoplasms"[Title/Abstract] OR "Neoplasms, Lung"[Title/Abstract] OR "Lung Neoplasm"[Title/Abstract] OR "Neoplasm, Lung"[Title/Abstract] OR "Neoplasms, Pulmonary"[Title/Abstract] OR "Neoplasm, Pulmonary"[Title/Abstract] OR "Pulmonary Neoplasm"[Title/Abstract] OR "Lung Cancer"[Title/Abstract] OR "Cancer, Lung"[Title/Abstract] OR "Cancers, Lung"[Title/Abstract] OR "Lung Cancers"[Title/Abstract] OR "Pulmonary Cancer"[Title/Abstract] OR "Cancer, Pulmonary"[Title/Abstract] OR "Cancers, Pulmonary"[Title/Abstract] OR "Pulmonary Cancers"[Title/Abstract] OR "Cancer of the Lung"[Title/Abstract] OR "Cancer of Lung"[Title/Abstract]))) OR ("Lung Tumor"))) AND ((Gefitinib[MeSH Terms]) OR (Gefitinib[Title/Abstract] OR "N-(3-Chloro-4-fluorophenyl)-7-methoxy-6-(3-(4-morpholinyl)propoxy)-4-quinazolinamide"[Title/Abstract] OR Iressa[Title/Abstract] OR ZD1839[Title/Abstract] OR "ZD 1839"[Title/Abstract])) Filters: English, Portuguese, Spanish Sort by: Publication Date	
BVS/BIREME	((micrornas OR "MicroRNA" OR mirnas OR "Micro RNA" OR "RNA, Micro" OR mirna OR "Primary MicroRNA" OR "MicroRNA, Primary" OR "Primary miRNA" OR "miRNA, Primary" OR "pri-miRNA" OR "pri miRNA" OR "RNA, Small Temporal" OR "Temporal RNA, Small" OR strna OR "Small Temporal RNA" OR "pre-miRNA" OR "pre miRNA") OR (epigenomics OR epigenomic OR epigenetics OR epigenetic) OR ("Polymorphism, Single Nucleotide" OR "Nucleotide Polymorphism, Single" OR "Nucleotide Polymorphisms, Single" OR "Polymorphisms, Single Nucleotide" OR "Single Nucleotide Polymorphisms" OR "SNPs" OR "Single Nucleotide Polymorphism") OR ("Polymorphism, Genetic" OR "Polymorphisms, Genetic" OR "Genetic Polymorphisms" OR "Genetic Polymorphism" OR "Polymorphism (Genetics)" OR "Polymorphisms (Genetics)")) AND (("Carcinoma, Non-Small-Cell Lung" OR "Carcinoma, Non Small Cell Lung" OR "Carcinomas, Non-Small-Cell Lung" OR "Lung Carcinoma,	267

	Non-Small-Cell" OR "Lung Carcinomas, Non-Small-Cell" OR "Non-Small-Cell Lung Carcinomas" OR "Nonsmall Cell Lung Cancer" OR "Non-Small-Cell Lung Carcinoma" OR "Non Small Cell Lung Carcinoma" OR "Carcinoma, Non-Small Cell Lung" OR "Non-Small Cell Lung Cancer") OR ("Lung Neoplasms" OR "Pulmonary Neoplasms" OR "Neoplasms, Lung" OR "Lung Neoplasm" OR "Neoplasm, Lung" OR "Neoplasms, Pulmonary" OR "Neoplasm, Pulmonary" OR "Pulmonary Neoplasm" OR "Lung Cancer" OR "Cancer, Lung" OR "Cancers, Lung" OR "Lung Cancers" OR "Pulmonary Cancer" OR "Cancer, Pulmonary" OR "Cancers, Pulmonary" OR "Pulmonary Cancers" OR "Cancer of the Lung" OR "Cancer of Lung") OR ("Lung Tumor")) AND ((gefitinib OR "N-(3-Chloro-4-fluorophenyl)-7-methoxy-6-(3-(4-morpholinyl)propoxy)-4-quinazolinamide" OR iressa OR zd1839 OR "ZD 1839")) AND (la:("en" OR "es"))	
WEB OF SCIENCE	TÓPICO: (MicroRNAs OR "MicroRNA" OR miRNAs OR "Micro RNA" OR "RNA, Micro" OR miRNA OR "Primary MicroRNA" OR "MicroRNA, Primary" OR "Primary miRNA" OR "miRNA, Primary" OR "pri-miRNA" OR "pri miRNA" OR "RNA, Small Temporal" OR "Temporal RNA, Small" OR stRNA OR "Small Temporal RNA" OR "pre-miRNA" OR "pre miRNA")<br>OR TÓPICO: (Epigenomics OR Epigenomic OR Epigenetics OR Epigenetic) OR TÓPICO: ("Polymorphism, Single Nucleotide" OR "Nucleotide Polymorphism, Single" OR "Nucleotide Polymorphisms, Single" OR "Polymorphisms, Single Nucleotide" OR "Single Nucleotide Polymorphisms" OR "SNPs" OR "Single Nucleotide Polymorphism") OR TÓPICO: ("Polymorphism, Genetic" OR "Polymorphisms, Genetic" OR "Genetic Polymorphisms" OR "Genetic Polymorphism" OR "Polymorphism (Genetics)<br>" OR "Polymorphisms (Genetics) ") Índices=SCI-EXPANDED, SSCI, A&HCI, CPCI-S, CPCI-SSH, ESCI Tempo estipulado=Todos os anos AND TÓPICO: ("Carcinoma, Non-Small-Cell Lung" OR "Carcinoma, Non Small Cell Lung" OR "Carcinomas, Non-Small-Cell Lung" OR "Lung Carcinoma, Non-Small-Cell" OR "Lung Carcinomas, Non-Small-Cell" OR "Non-Small-Cell Lung Carcinomas" OR "Nonsmall Cell Lung Cancer" OR "Non-Small-Cell Lung Carcinoma" OR "Non Small Cell Lung Carcinoma" OR "Carcinoma, Non-Small Cell Lung" OR "Non-Small Cell Lung Cancer") OR TÓPICO: ("Lung Neoplasms" OR "Pulmonary Neoplasms" OR "Neoplasms, Lung" OR "Lung Neoplasm" OR "Neoplasm, Lung" OR "Neoplasms, Pulmonary" OR "Neoplasm, Pulmonary" OR "Pulmonary Neoplasm" OR "Lung Cancer" OR "Cancer, Lung" OR "Cancers, Lung" OR "Lung Cancers" OR "Pulmonary Cancer" OR "Cancer, Pulmonary" OR "Cancers, Pulmonary" OR "Pulmonary Cancers" OR "Cancer of the Lung" OR "Cancer of Lung") OR	431

	Todos os campos: ("Lung Tumor") Índices=SCI-EXPANDED, SSCI, A&HCI, CPCI-S, CPCI-SSH, ESCI Tempo estipulado=Todos os anos AND TÓPICO: (Gefitinib OR "N-(3-Chloro-4-fluorophenyl) -7-methoxy-6-(3-(4-morpholinyl)propoxy)-4-quinazolinamide" OR Iressa OR ZD1839 OR "ZD 1839") Índices=SCI-EXPANDED, SSCI, A&HCI, CPCI-S, CPCI-SSH, ESCI Tempo estipulado=Todos os anos AND Refinado por: IDIOMAS: (ENGLISH)Índices=SCI-EXPANDED, SSCI, A&HCI, CPCI-S, CPCI-SSH, SCI Tempo estipulado=Todos os anos	
EMBASE	('microrna'/exp OR 'microrna'/syn OR 'epigenetics'/exp OR 'epigenetics'/syn OR 'single nucleotide polymorphism'/exp OR 'single nucleotide polymorphism'/syn OR 'genetic polymorphism'/exp OR 'genetic polymorphism'/syn) AND ('non small cell lung cancer'/exp OR 'non small cell lung cancer'/syn OR 'lung tumor'/exp OR 'lung tumor'/syn) AND ('gefitinib'/exp OR 'gefitinib'/syn) AND ([english]/lim OR [portuguese]/lim OR [spanish]/lim) AND [embase]/lim NOT ([embase]/lim AND [medline]/lim)	442
COCHRANE LIBRARY	MeSH descriptor: [MicroRNAs] explode all trees OR (MicroRNAs OR "MicroRNA" OR miRNAs OR "Micro RNA" OR "RNA, Micro" OR miRNA OR "Primary MicroRNA" OR "MicroRNA, Primary" OR "Primary miRNA" OR "miRNA, Primary" OR "pri-miRNA" OR "pri miRNA" OR "RNA, Small Temporal" OR "Temporal RNA, Small" OR stRNA OR "Small Temporal RNA" OR "pre-miRNA" OR "pre miRNA"):ti,ab,kw OR MeSH descriptor: [Epigenomics] in all MeSH products OR (Epigenomics OR Epigenomic OR Epigenetics OR Epigenetic):ti,ab,kw OR MeSH descriptor: [Polymorphism, Single Nucleotide] explode all trees OR ("Polymorphism, Single Nucleotide" OR "Nucleotide Polymorphism, Single" OR "Nucleotide Polymorphisms, Single" OR "Polymorphisms, Single Nucleotide" OR "Single Nucleotide Polymorphisms" OR "SNPs" OR "Single Nucleotide Polymorphism"):ti,ab,kw OR MeSH descriptor: [Polymorphism, Genetic] explode all trees OR ("Polymorphism, Genetic" OR "Polymorphisms, Genetic" OR "Genetic Polymorphisms" OR "Genetic Polymorphism" OR "Polymorphism (Genetics)" OR "Polymorphisms (Genetics)"):ti,ab,kw AND MeSH descriptor: [Carcinoma, Non-Small-Cell Lung] explode all trees OR ("Carcinoma, Non-Small-Cell Lung" OR "Carcinoma, Non Small Cell Lung" OR "Carcinomas, Non-Small-Cell Lung" OR "Lung Carcinoma, Non-Small-Cell" OR "Lung Carcinomas, Non-Small-Cell" OR "Non-Small-Cell Lung Carcinomas" OR "Nonsmall Cell Lung Cancer" OR "Non-Small-Cell Lung Carcinoma" OR "Non Small Cell Lung Carcinoma" OR "Carcinoma, Non-Small Cell Lung" OR "Non-Small Cell Lung Cancer"):ti,ab,kw OR MeSH descriptor: [Lung Neoplasms]	05

	explode all trees OR ("Lung Neoplasms" OR "Pulmonary Neoplasms" OR "Neoplasms, Lung" OR "Lung Neoplasm" OR "Neoplasm, Lung" OR "Neoplasms, Pulmonary" OR "Neoplasm, Pulmonary" OR "Pulmonary Neoplasm" OR "Lung Cancer" OR "Cancer, Lung" OR "Cancers, Lung" OR "Lung Cancers" OR "Pulmonary Cancer" OR "Cancer, Pulmonary" OR "Cancers, Pulmonary" OR "Pulmonary Cancers" OR "Cancer of the Lung" OR "Cancer of Lung"):ti,ab,kw OR ("Lung Tumor") AND MeSH descriptor: [Gefitinib] explode all trees OR (Gefitinib OR "N-(3-Chloro-4-fluorophenyl)-7-methoxy-6-(3-(4-morpholinyl)propoxy)-4-quinazolinamide" OR Iressa OR ZD1839 OR "ZD 1839"):ti,ab,kw	
